# Self-reported medication information needs among medication users in a general population aged 40 years and above – the Tromsø study

**DOI:** 10.1186/s12889-022-14573-z

**Published:** 2022-11-25

**Authors:** Mari J. Walquist, Kristian Svendsen, Beate H. Garcia, Trine S. Bergmo, Anne Elise Eggen, Kjell H. Halvorsen, Lars Småbrekke, Unni Ringberg, Elin C. Lehnbom, Marit Waaseth

**Affiliations:** 1 Department of Pharmacy, Faculty of Health Sciences, UiT The Arctic University of Norway, Tromsø, Norway; 2 Norwegian College of Fishery Science, The Faculty of biosciences, fisheries and economics, UiT The Arctic University of Norway, Tromsø, Norway; 3 Norwegian Centre for E-Health Research, University Hospital of North Norway, Tromsø, Norway; 4 Department of Community Medicine, Faculty of Health Sciences, UiT The Arctic University of Norway, Tromsø, Norway

**Keywords:** Health survey, Population based, Medication information need, Information sources, Medication adherence, Health anxiety

## Abstract

**Purpose:**

To determine the prevalence and associated factors of self-reported medication information needs among medication users in a general population aged 40 years and above – The Tromsø Study.

**Methods:**

Cross-sectional study of medication users (*n* = 10,231) among participants in the Tromsø Study, a descriptive analysis of questionnaire data and multivariable logistic regression (*n* = 9,194).

**Results:**

Sixteen percent of medication users expressed a need for more information about own medications. Overall, medication users agreed to a higher degree to have received information from the GP compared to the pharmacy. Concerned medication users and those disagreeing to have received information about side effects had the highest odds for needing more information (OR 5.07, 95% CI 4.43–5.81) and (OR 2.21, 95% CI 1.83–2.68), respectively. Medication users who used heart medications (e.g., nitroglycerin, antiarrhythmics, anticoagulants) (OR 1.71, 95% CI 1.46–2.01), medication for hypothyroidism (OR 1.36, 95% CI 1.13–1.64) or had moderately health anxiety had expressed need for medication information. Whereas medication users with lower education, those that never used internet to search for health advice, and medication users who disagreed to have received information about reason-for-use were associated with lower odds (OR 0.75, 95% CI 0.62–0.91), (OR 0.85, 95% CI 0.74–0.98) and (OR 0.68, 95% CI 0.53–0.88), respectively.

**Conclusion:**

This study demonstrated that there is need for more information about own medications in a general population aged 40 years and above and shed light on several characteristics of medication users with expressed information need which is important when tailoring the right information to the right person.

## Introduction

Medications play a substantial role in management of people’s health. Effective systems of medication information can contribute to optimal medication use and enable medication users to make informed choices about their treatment [1]. The main sources of medication information are general practitioners (GP) or other health care providers including community pharmacy staff, the Internet and medication leaflets [2–4]. People develop habits for information seeking and tend to revisit the same helpful information sources as used in the past [5]. Despite the essentially unlimited access to information, patients report dissatisfaction with received medication information [6–10]. Overestimation of patients` knowledge about their illness and understanding of medication regimen may lead to suboptimal communication between patients and health care providers [11]. In addition, low health literacy has been associated with under-diagnosis and under-treatment, but also as a potential driver for over-diagnosis and over-treatment [12]. Since medication information need is personal, shown to vary over time and related to diagnosis it is important to tailor given information [13, 14]. Also, common to most patients is the importance of a good relationship with health care personnel and that the information is given in an accessible, timely manner [15]. However, as shown in a review by Clarke et al., limited access to the GP leads patients to use the Internet to access information, despite trusting their GP more due to clinical expertise and experience [5]. This review included articles from several countries that may be more or less comparable to Norway. However, since Norwegian patients must prebook time with the GP with a variation of waiting time, the Norwegian medication users may also use the Internet to access information instead of the GP.

Unmet medication information needs are a barrier to medication adherence [16]. However, it is commonly more manageable than beliefs and other perceptual factors influencing adherence [17]. Concerns about side effects have been identified as a key factor to non-adherence related to how patients individually weigh the perceived risks of medication against benefits [18]. Characteristics previously reported to be related to need for more medication information include age, socio-economic status, and disease [13, 19] along with an intrinsic desire for health information and worries about changes in medications [20]. In order to design intervention to provide medication information, it is necessary to identify populations with most need for information and identify which type of information is needed. Due to cultural differences and geographical variation in treatment traditions and health care, the global generalizability of the research literature in this field is uncertain. To what extent the above-mentioned factors and characteristics are associated with medication information needs in Norway or other relatable Nordic countries remains to be investigated.

Consequently, this study aimed to determine the prevalence and associated factors of self-reported medication information needs among medication users in a general population 40 years and above—the Tromsø Study, Norway.

## Material and methods

### Study design

This is a cross-sectional study with self-reported data from the Tromsø Study, which is a population-based health study in the municipality of Tromsø, Norway. The Tromsø Study includes seven waves of data collections conducted between 1974 and 2016. Data are collected through questionnaires, health examinations and blood samples. We included participants from Tromsø7 (2015–2016). Tromsø7 is a multipurpose population-based health survey and the details of study design, data collection, attendance, and prevalence of risk factors and disease are described previously by Hopstock et al., [21]. In Tromsø7 all inhabitants aged 40 years or older (*n* = 32,591) were invited, and 21,083 (65%) participated [21].

### Study population

**Fig. 1:**
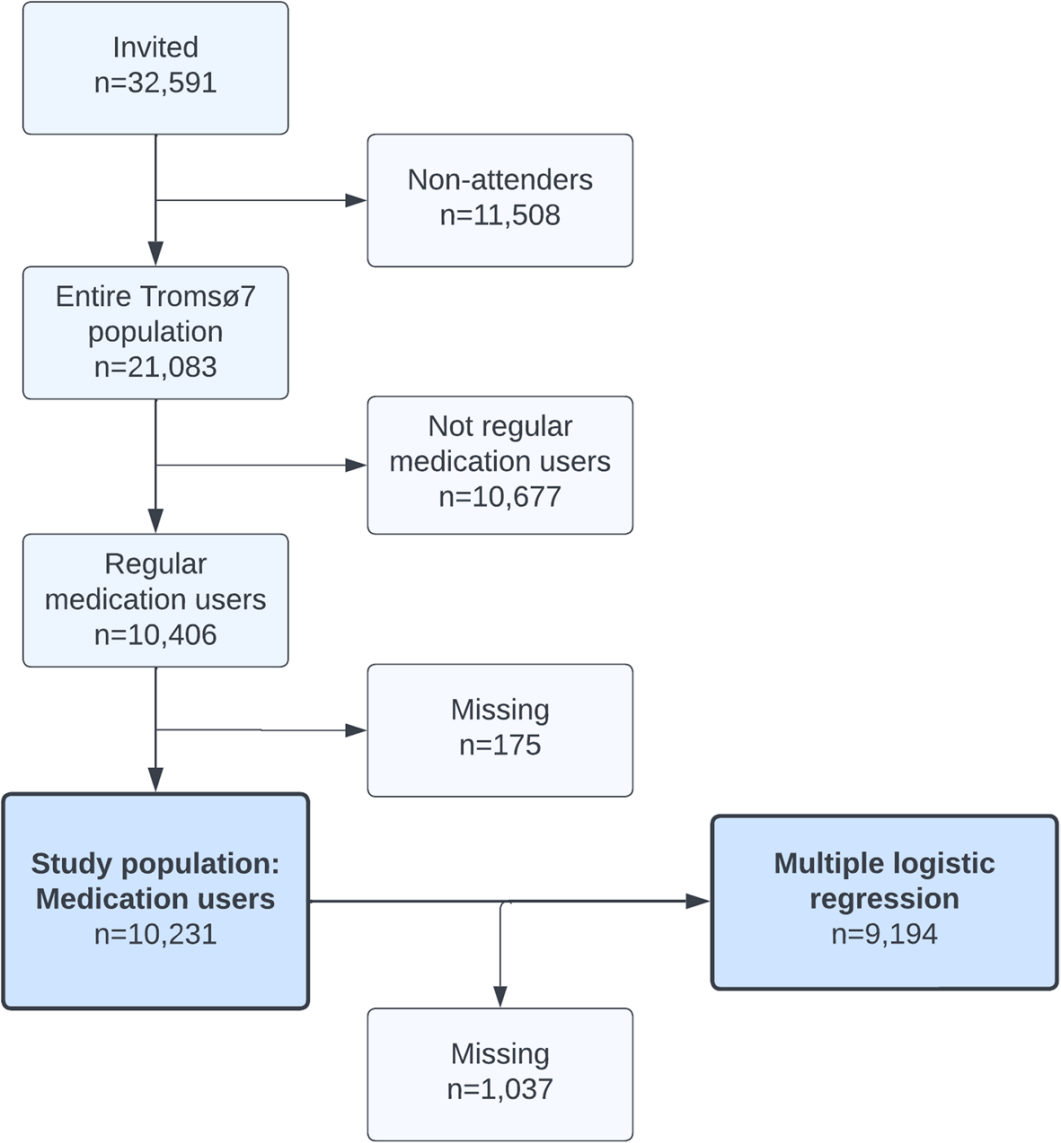
Flow chart of the study population. Regular medication users used prescription or non-prescription medications the last four weeks. Study population of medication users were participants with non-missing dependent variable, i.e., the need for more information. Multiple logistic regression population represent medication users with non-missing values on all variables included in the analysis. Figure created at lucidchart.com

We included participants who answered “yes” to regular use of medications during the last four weeks, prescription or non-prescription (*n* = 10,406, 49%) in our study. Participants with missing answer to the variable “*Do you need more information about your medications?*” were excluded (*n* = 175). Consequently, the final study population of medication users comprised 10,231 participants (Fig. 1).

### Variables

In an explorative approach, our set of variables was decided based on 1) factors and characteristics identified in previous research and 2) other factors that might have an influence on medication information need based on the research group`s joint experience from communication with medication users, but 3) limited to the data available from Tromsø7. The following variables were included in our analyses: demography (sex and age), socio-economic status (education and work situation) and health (self-reported health status, worry about own health or medication, health anxiety, medication use, use of health services and online services for advice on health and disease issues, and received medication information from health personnel [GP and pharmacy]), see Tables 1, 2 and 3 for details on response alternatives. The questionnaires and a detailed description of all variables in Tromsø7 are accessible from the Tromsø Study webpage [22]. To define the proportion in need of more medication information, we used the variable *“Do you need more information about your medications?”, which was included for the first time in Tromsø7.* We dichotomized the variable by combining the answers “I need some more information” and “I need a lot more information” into “Yes” and keeping “I do not need any more information” as “No”. Prior to the statistical analysis, several variables (Table 4) were aggregated as following; Health status was aggregated into “Bad” (very bad and bad), “Neither bad nor good” and “Good” (good and excellent). Low faith in doctors and worry about own health were aggregated into “Not at all”, “Moderately” (a little bit and moderately) and “A great deal” (quite a bit and a great deal). Anxious and depressed was aggregated into “Not at all”, “Moderately” (slightly and moderately) and “A great deal” (severely and extremely). Concerned about own medications was aggregated into “Not at all” and “Concerned” (concerned and very concerned). Medicines for heart disease (e.g., nitroglycerin, antiarrhythmics, anticoagulants) and medications for hypothyroidism were aggregated into “Yes” (current use) and “No” (previously not currently and never used). Internet health advice comprise four variables (apps, search engines, social media and video services shown in Table 2) and was aggregated into one new variable with two categories “Never” and “Used” (once, a few times and often). Agreement with statements regarding received medication information were aggregated into “Agree” (strongly agree GP and pharmacy, agree GP and pharmacy, strongly agree GP or pharmacy, and agree GP or pharmacy), “Neither agree nor disagree” (GP and pharmacy) and “Disagree” (strongly disagree GP and pharmacy, disagree GP and pharmacy, strongly disagree GP or pharmacy and disagree GP or pharmacy). The variables were aggregated because the focus was on a potential association between information need and having received information from health personnel (GP and pharmacy). The degree of agreement will be explored further in a subsequent paper in addition to potential variation related to information sources and specific medications used.

**Table 1:** Demographic, socio-economic and health characteristics of medication users (n and %)

	**Medication users (*n* = 10,231)**	
	**n**	**%**
Sex		
Female	5,543	54.2
Male	4,688	45.8
Age Groups		
40–49	2,325	22.7
50–59	2,726	26.2
60–69	2,871	28.1
70–79	1,767	17.3
80 +	542	5.3
Education		
Primary School	2,715	26.5
Upper secondary education	2,822	27.6
College/University < 4 years	1,949	19.0
College/University ≥ 4 years	2,522	24.7
Work situation		
Works full time	4,675	45.7
Works part time	886	8.7
Unemployed	63	0.6
Housekeeping	74	0.7
Disability benefit recipient or work assessment allowance	1,304	12.7
Family income supplement	15	0.1
Retired	3,162	30.9
Student/military	27	0.3
Health status		
Very bad	55	0.5
Bad	808	7.9
Neither bad nor good	3,414	33.4
Good	5,021	49.1
Excellent	832	8.1
*Visited the last 12 months?*		
General practitioner		
No	981	9.6
Yes	9,171	89.6
Pharmacy		
No	1,526	14.9
Yes	8,329	81.4
Low faith in doctor		
Not at all	6,928	67.7
A little bit	2,277	22.3
Moderately	578	5.6
Quite a bit	162	1.6
A great deal	76	0.7
Worry about health		
Not at all	3,534	34.5
A little bit	4,671	45.7
Moderately	1,298	12.7
Quite a bit	453	4.4
A great deal	109	1.1
Anxious or depressed		
No	7,549	73.8
Slightly	1,992	19.5
Moderately	452	4.4
Severely	118	1.2
Extremely	16	0.2

**Table 2:** Medication related characteristics of medication users (n and %)

	**Medication users (*n* = 10,231)**	
	**n**	**%**
In need of more information about own medications		
Yes	1,637	16.0
No	8,594	84.0
Number of medications		
0	1,095	10.7
1–4	7,200	70.4
5–8	1,702	16.6
9–12	207	2.0
≥ 13	27	0.3
Importance of own medications		
Not important at all	227	2.2
Not very important	701	6.9
Important	4,833	47.2
Very important	4,414	43.1
Concern about own medications		
Not at all concerned	8,141	79.6
Concerned	1,937	18.9
Very concerned	97	0.9
Blood pressure lowering medications		
Currently	3,716	36.3
Previously, not now	333	3.3
Never used	5,989	58.5
Cholesterol lowering medications		
Currently	2,524	24.7
Previously, not now	409	4.0
Never used	7,023	68.6
Medications for heart disease^a^		
Currently	2,029	19.8
Previously, not now	270	2.6
Never used	7,544	73.7
Insulin		
Currently	243	2.4
Previously, not now	49	0.5
Never used	9,538	93.5
Tablets for diabetes		
Currently	569	5.6
Previously, not now	89	0.9
Never used	9,228	90.2
Medications for hypothyroidism		
Currently	1,147	11.2
Previously, not now	85	0.8
Never used	8,647	84.5
Diuretics		
Currently	709	6.9
Previously, not now	411	4.0
Never used	8,655	84.6
*Use of online services for advice on health and disease issues:*		
Applications (apps)		
Once	204	2.0
A few times	912	8.9
Often	174	1.7
Never	8,518	83.3
Search engines (e.g., Google)		
Once	769	7.5
A few times	3,473	33.9
Often	551	5.4
Never	5,191	50.7
Social media (e.g., Facebook)		
Once	123	1.2
A few times	474	4.6
Often	161	1.6
Never	8,820	86.2
Video services (e.g., YouTube)		
Once	110	1.2
A few times	309	3.2
Often	48	0.5
Never	9,081	95.1

*GP* General practitioner

^a^E.g., nitroglycerin, antiarrhythmics, anticoagulants

**Table 3:** Medication information received from health personnel (n and %)

Questionnaire: *Think about the information you receive about your medications from your GP/pharmacy. Indicate whether you agree that you receive the following information:*		GP	Pharmacy	GP %	Pharmacy %
Reason for taking my medications	Strongly agree	6,145	3,248	60.1	31.7
	Agree	3,096	3,124	30.3	30.5
	Neither agree nor disagree	412	1,342	4.0	13.1
	Disagree	127	1,154	1.2	11.3
	Strongly disagree	280	1,113	2.7	10.9
How to use my medications	Strongly agree	5,808	3,932	56.8	38.4
	Agree	3,166	3,884	30.9	38.0
	Neither agree nor disagree	496	956	4.8	9.3
	Disagree	219	581	2.1	5.7
	Strongly disagree	306	647	3.0	6.0
Side effects I should be aware of	Strongly agree	3,064	2,525	29.9	24.7
	Agree	2,661	2,761	26.0	27.0
	Neither agree nor disagree	2,526	2,402	24.7	23.5
	Disagree	1,053	1,289	10.3	12.6
	Strongly disagree	675	979	6.6	9.6
Interactions – the influence from food and other medications	Strongly agree	2,542	2,313	24.8	22.6
	Agree	2,156	2,367	21.1	23.1
	Neither agree nor disagree	2,970	2,662	29.0	26.0
	Disagree	1,275	1,370	12.5	13.4
	Strongly disagree	973	1,182	9.5	11.6

*GP* General practitioner

**Table 4:** Characteristics associated with expressing a need for more information about own medications. (Multiple Logistic Regression, *n* = 9,194)

Variables	Categories	n	SLR	MLR
			**OR (95% CI)**	**OR (95% CI)**
Age Groups^a^	40–49	2,236	1.0	1.0
	50–59	2,547	0.96 (0.83–1.12)	0.96 (0.80–1.14)
	60–69	2,563	0.96 (0.83–1.11)	0.98 (0.81–1.17)
	70–79	1,474	0.95 (0.80–1.12)	1.06 (0.84–1.32)
	80 +	374	0.99 (0.77–1.27)	1.21 (0.86–1.71)
Education	Primary School	2,343	0.85 (0.73–0.99)^*^	0.75 (0.62–0.91)^*^
	Upper secondary education	2,589	0.91 (0.79–1.06)	0.86 (0.73–1.02)
	College/University < 4 years	1,846	1.07 (0.92–1.25)	0.94 (0.79–1.13)
	College/University ≥ 4 years	2,416	1.0	1.0
Health status^b^	Bad	746	2.53 (2.12–3.00)^*^	1.11 (0.88–1.40)
	Neither bad nor good	3,015	1.95 (1.74–2.18)^*^	1.34 (1.16–1.55)^*^
	Good	5,433	1.0	1.0
Low faith in doctor^c^	Not at all	6,387	1.0	1.0
	Moderately	2,591	2.61 (2.33–2.92)^*^	1.57 (1.36–1.81)^*^
	A great deal	216	3.67 (2.77–4.87)^*^	**1.83 (1.29–2.59)**
Worry about own health^c^	Not at all	3,253	1.0	1.0
	Moderately	5,453	2.48 (2.17–2.84)^*^	1.25 (1.06–1.47)^*^
	A great deal	488	4.60 (3.72–5.71)^*^	1.30 (0.95–1.74)
Anxious or depressed^d^	Not at all	6,877	1.0	1.0
	Moderately	2,198	2.02 (1.80–2.26)^*^	1.17 (1.02–1.36)^*^
	A great deal	119	1.70 (1.12–2.60)^*^	0.81 (0.49–1.35)
Concerned about own medications^e^	Not at all	7,372	1.0	1.0
	Concerned	1,822	**6.40 (5.71–7.18)**	5.07 (4.43–5.81)^*^
Medications for heart disease^f^	Yes	1,774	1.73 (1.53–1.95)^*^	1.71 (1.46–2.01)^*^
	No	7420	1.0	1.0
Medications for hypothyroidism^f^	Yes	1,007	1.33 (1.14–1.57)^*^	1.36 (1.13–1.64)^*^
	No	8,187		1.0
Internet health advice^g^	Used	4,883	1.0	1.0
	Never	4,311	0.72 (0.65–0.80)^*^	0.85 (0.74–0.98)^*^
Information: reason for use^h^	Agree	8,488	1.0	1.0
	Neither agree nor disagree	140	2.70 (1.94–3.80)^*^	1.14 (0.75–1.74)
	Disagree	566	1.34 (1.10–1.63)^*^	0.68 (0.53–0.88)^*^
Information: side effects^h^	Agree	6,137	1.0	1.0
	Neither agree nor disagree	1,155	3.46 (2.99–4.00)^*^	2.52 (2.06–3.09)^*^
	Disagree	1,902	3.16 (2.79–3.58)^*^	2.21 (1.83–2.68)^*^
Information: interactions^h^	Agree	5,206	1.0	1.0
	Neither agree nor disagree	1,482	2.74 (2.38–3.17)^*^	1.56 (1.28–1.91)^*^
	Disagree	2,506	2.92 (2.59–3.30)^*^	1.75 (1.46–2.11)^*^

Model summary: Omnibus test (Chi-square = 1513, df = 25, significant   < 0.001), Nagelkerke R^2^ (0.261), Hosmers & Lemenshow test (Chi-Square = 8.32, df = 8, significant = 0.403). Overall prediction = 85.7%. ^*^Significant *p* < 0.05

^b^Age groups are not associated with expressed need for information, however, this insignificant variable was included in the regression model to adjust for age

^b^Health status was aggregated into “Bad” (very bad and bad), “Neither bad nor good” and “Good” (good and excellent)

^c^Low faith in doctors and worry about own health were aggregated into “Not at all”, “Moderately” (a little bit and moderately) and “A great deal” (quite a bit and a great deal)

^d^Anxious and depressed was aggregated into “Not at all”, “Moderately” (slightly and moderately) and “A great deal” (severely and extremely)

^e^Concerned about own medications was aggregated into “Not at all” and “Concerned” (concerned and very concerned)

^f^Medicines for heart disease (e.g., nitroglycerin, antiarrhythmics, anticoagulants) and medications for hypothyroidism were aggregated into “Yes” (current use) and “No” (previously not currently and never used)

^g^Internet health advice comprise four variables (apps, search engines, social media and video services shown in Table 2) and was aggregated into one new variable with two categories “Never” and “Used” (once, a few times and often)

^h^Information variables were aggregated into “Agree” (strongly agree GP and pharmacy, agree GP and pharmacy, strongly agree GP or pharmacy and agree GP or pharmacy), “Neither agree nor disagree GP and pharmacy) and “Disagree” (strongly disagree GP and pharmacy, disagree GP and pharmacy, strongly disagree GP or pharmacy and disagree GP or pharmacy)

*GP* General practitioner, *SLR *Simple logistic regression, *MLR *Multiple logistic regression

### Statistics

We have used IBM SPSS (Statistical Package for the Social Sciences) for Macintosh, version 28.0 (IBM Corp., Armonk, NY, US) for the statistical analyses. Descriptive statistics includes frequencies and percentages. To identify characteristics associated with need for information about own medications (dependent variable) we conducted Multiple Logistic Regression (MLR). The results are presented as odds ratios (OR) with 95% confidence intervals (CI) and only medication users with non-missing values on all variables were included in the MLR analysis. All variables in Tables 1, 2 and 3 were initially included in the MLR analysis. Subsequently, non-significant variables (*p* > 0.05) were excluded through stepwise backward elimination. The final MLR model with significant variables and reference categories is shown in Table 4 and comprise 9,194 medication users (Fig. 1). The insignificant variable “age groups” was included in the MLR analysis to ensure that the final model was adjusted for age. The MLR population (*n* = 9,194) and the medication user population (*n* = 10,231) were compared by descriptive statistics (frequencies) and no noteworthy differences were uncovered. We also conducted a Simple Logistic Regression (SLR) for each explanatory variable for comparison (Table 4).

## Results

The study population included 10,231 medication users and comprised 54.2% women. Demographic and socio-economic information together with health characteristics such as age and health status are summarized in Table 1. The most frequently used medications were blood pressure lowering medication (36.3%), cholesterol lowering medication (24.7%) and other medications for heart disease, e.g., nitroglycerin, antiarrhythmics, anticoagulants (19.8%). Furthermore, 79.6% of medication users were not concerned about own medications (Table 2).

In total, 16% of medication users reported unmet information needs regarding own medications. Most medication users had never used online services (such as apps, search engines, social media, or video services) for seeking advice on health and disease issues (83.3%, 50.7%, 86.2% and 95.1%, respectively) (Table 2). Medication users agreed to have received information about why and how to take medications from their GP (90.4% and 87.7%, respectively) and pharmacy (62.2% and 76.4%, respectively) (Table 3). However, medication users agreed to a lesser extent to have received information about side effects and interactions from their GP (56% and 46%, respectively) and pharmacy (51.7% and 45.7%, respectively).

Characteristics associated with information need are summarized in Table 4. Lower education was associated with lower odds of needing information compared with the highest education, although significant only for primary school (OR = 0.75, 95% CI = 0.62–0.91). Regarding health-related variables, medication users considering their own health to be neither bad nor good had higher odds for needing medications information compared to medication users with perceived good health (OR = 1.34, 95% CI = 1.16–1.55). Regarding the low faith in doctor, “moderately” and “a great deal”, were positively associated with higher information need compared to medication users reporting “not at all” (OR = 1.57, 95% CI = 1.36–1.81 and [OR = 1.83, 95% CI = 1.29–2.59, respectively). This similarly applies to the other health anxiety variables; moderate worry about own health (OR = 1.25, 95% CI = 1.06–1.47) and moderately anxious or depressed (OR = 1.17, 95% CI = 1.02–1.36). Being concerned about own medications produced the strongest association with needing information about own medications (OR = 5.07, 95% CI = 4.43–5.81). Current use of heart medications (e.g., nitroglycerin, antiarrhythmics, anticoagulants) and medications for hypothyroidism were positively associated with information need compared with medication users that never used these particular medications (OR = 1.71, 95% CI = 1.46–2.01) and (OR = 1.36, 95% CI = 1.13–1.64), respectively. Medication users who had never used the internet in seeking health advice had lower odds for information need (OR = 0.85, 95% CI = 0.74–0.98). Regarding the four statements about received medication information from health care providers (GP or pharmacy), the reference category was medication users who agreed to have received such information. Medication users answering neutral or who disagreed to have received information about side effects and interactions had higher odds for information need when compared with the reference category (OR = 2.52, 95% CI = 2.06–3.09), (OR = 2.21, 95% CI = 1.83–2.68), (OR = 1.56, 95% CI = 1.28–1.91) and (OR = 1.75, 95% CI = 1.46–2.11), respectively. Regarding agreement to having received information about reason-for-use of medication, compared with the reference category (agree), medication users who disagreed to have received information had lower odds of needing medication information (OR = 0.68, 95% CI = 0.53–0.88).

## Discussion

In this study we found that 16% of medication users in a general adult population aged 40 years and above expressed a need for more information about their own medications. We also identified several characteristics associated with unmet information need and the strongest associations were for concerns about own medications and disagreeing having received information about side effects or interactions from health care providers.

To our knowledge, there are few comparable studies investigating medication information need in a general adult population. Another Norwegian study reported that 56% of the respondents would like to know more about their medications [7]. This study was, however, small and included only elderly adults (*n* = 162, age ranged from 62 to 96 years). The group of medication users expressing unmet information need about their own medication may be seen in relation to the high percentage of medication users agreeing to have received information about their medications from their GP or pharmacy. Presumably, this group of medication users felt informed, regardless of their current level of medication knowledge. Especially information regarding why and how to take medications had a high agreement rate from the GP and, to a lesser extent, the pharmacy. It has been reported that medication users were satisfied with receiving a minimum of information and had no expectations of more information [15]. Giving medication information is important to contribute to knowledge about own medications. However, if not given correctly it may also defeat its purpose. This is illustrated in the study by Duggan et al., where patients who were satisfied with limited knowledge and who did not express a wish for more information, felt less empowered and more worried after being given additional information [20]. This emphasizes the importance of identifying the medication users interested in more information about own medications and avoid information overload for those not interested. A Finnish study showed a seven-fold increase in patients who stated that they did not receive medication information from any source from 1999–2014 [23]. As neighbouring countries with similar healthcare systems, this may also apply to Norway and indicate that an increasing proportion of the population is unaccustomed to receiving medication information which might lead to less expressed need. Overall, our respondents agreed to a higher degree that they have received information from GP compared to the pharmacy. This corresponds with previous literature indicating the GP as the preferred information source and emphasize that the pharmacy has an unfulfilled information potential [5, 7].

We identified several characteristics associated with unmet information need. Medication users who were concerned about their own medications had high odds for needing more medication information. Kusch et al. describes how concerns about medications is a central factor related to non-adherence, and how receiving too much unsolicited information or not enough information both may amplify concerns [24]. Therefore, health care providers should be attentive and attempt to unveil such concern among medication users and ensure that their medication information needs are met, and that information overload is prevented. The self-reported variable education is recently validated [25] and lower education (primary school) gave lower odds for expressing need for more information about own medications. Lower education has been associated with higher adherence [26] in addition to overestimation of own knowledge and understanding of received information [27] which may support this finding.

Importance of a trusting patient-doctor relationship has been thoroughly investigated and found to be essential for adherence [28]. Medication users with low faith in doctors had an expressed need for more information in our study. According to a literature review by Clarke et al., this may be related to the necessity of some patients to seek a second opinion after consultations to validate the received information and to explore alternative treatments [5]. Anxiety and depression are among the strongest predictors for non-adherence, making a huge impact on health, especially if the patient has chronic illnesses [11]. In our study, an unmet medication information need was expressed by those reporting to be moderately (but not severely) depressed or anxious, or worried about own health. Depression and anxiety disorders are the leading cause of disability worldwide [29], and consequently it is important to identify moderately depressed, anxious, and worried medication users and provide tailored medication information needed to prevent or limit disease progression. As physical attendance at the study site was mandatory for participation, we presume that invitees with severe mental illnesses attended to a lesser degree (< 1.5% in our study) since they are known to experience pessimisms, cognitive impairments, and withdrawal from social support [11]. The number of participants may consequently be too small to show a relationship between medications need in the severely anxious or depressed medication users.

Medication use investigated in this study comprises large medication groups such as blood pressure medications and other heart disease medications (e.g., nitroglycerin, antiarrhythmics, anticoagulants) along with more specific medications such as insulin and hypothyroidism medication. We found that current users of heart disease medications (e.g., nitroglycerin, antiarrhythmics, anticoagulants) and hypothyroidism medication had higher odds of needing more information than medication users not using these medications. The complex treatment regimens these medications often comprise may be confusing to the patient and lead to increased information need. Cardiac rehabilitation patients have previously reported that the greatest information needs have been related to medication, diagnosis, and treatments, together with emergency and safety [30]. Others have, on the other hand, reported that patients using cardiovascular medications had less expressed desire for information [13]. Hypothyroidism and information need have, to our knowledge, not previously been investigated and this unmet need may be underestimated by health care providers due to limited medication treatment alternatives. Still, hormone treatment is complex and require close individual follow-up to achieve optimum dosage as a fixed-dose treatment is infeasible [31]. At the same time, medication information need is shown to vary over time and related to patients experience with medication regimen [14] which increasing the intricacy of giving medication information.

Medication users who disagreed to have received information about side effects and interactions had higher odds of expressed need for more information. The strongest association was found for information on side effects. This corresponds with previous studies reporting that patients interested in more information mainly addressed matters about side effects and interactions [7, 8, 24]. A lower proportion medication users agreed to have received information about side effects and interactions from health care providers compared to information about how and why to take medications. Such information tends to be intricate and may represent a communication threshold compared to information on practical use and storage. Health care providers may also overestimate patients’ understanding or attentiveness [32]. Dissatisfied patients on information given from health care providers seek out information elsewhere to narrow information gaps [5]. At the same time, medication users who disagreed to have received information about reason-for-use had lower odds for information need. A complementary source of health information is the Internet, and the search for such information has increased tremendously the last decades [33–35]. Surprisingly, in our study, with data from 2015/16, more than half of medication users reported that they had never used any of the given internet sources (apps, search engines, social media, or video services) for seeking information or advice on health and disease issues. Interestingly, these medication users expressed less information need compared to those who had used these sources in a model adjusted for age. Patients not using internet sources for health advice have been shown to rely more on family and friends for health information and to trust their GP and prescribed treatment to an extended degree compared to those seeking such information online [5]. These medication users may also be unfamiliar with available internet sources or rather find it frustrating to obtain desired information online [2]. Consequently, the use of internet sources does not explain the low need for medication information in our study population.

The main strength of our study is the use data from a well-established, population-based health survey (Tromsø7). Tromsø7 invited all inhabitants ≥ 40 years in Tromsø municipality (Norway) and an acceptable attendance rate (65%) provided a large study sample of 10,231 medication users. One in ten did not report any use of medication although they answered yes to regular use of medication during the last four weeks and were thereby included in the study population. These may be non-current or non-regular users who have misinterpreted the question, or they do not want to disclose their medication use. We chose to include these as they may be in need of medicines information. The main limitation is that all health and socio-economic variables were self-reported, however, some variables have been validated (e.g., the education variable [25]). It is important to emphasize that physical attendance was required for inclusion in the study, which will limit the generalizability to inhabitants who are unable to physically attend the study site. The cross-sectional design does not allow for any conclusions regarding a causal relationship between the examined characteristics and medication information needs.

## Conclusions

This study showed that there is a need for more information about own medications in a general population aged above 40 years. Overall, in our study, medication users agreed that they to a higher degree had received information from GP compared to the pharmacy, which indicate that the pharmacy has an unfulfilled information potential. Our study shed light on several characteristics of medication users with expressed information need, such as concerned medication users and those with moderately health anxiety, which is important when tailoring the right information to the right person. On the other hand, medication users, such as, those with lower education and those not accustomed to use internet sources for seeking health advice, expressed less need for information, and health personnel should be careful not to cause information overload.

## Data Availability

Data used in this study are licensed and there are restrictions applied to the availability of the data. However, the data is available from the corresponding author on reasonable request and with permission from the Tromsø Study and the Regional Committees for Medical and Health Research Ethics.
